# 2-[(*E*)-3,4-Dimeth­oxy­benzyl­idene]hydrazinecarboxamide

**DOI:** 10.1107/S1600536812020739

**Published:** 2012-05-16

**Authors:** M. Nawaz Tahir, M. Naveed Umar, Akbar Ali, Hazoor Ahmad Shad

**Affiliations:** a University of Sargodha, Department of Physics, Sargodha, Pakistan; b Department of Chemistry, University of Malakand, Pakistan; c Department of Chemistry, Government Post Graduate College, Gojra, Punjab, Pakistan

## Abstract

In the title compound, C_10_H_13_N_3_O_3_, the 3,4-dimeth­oxy­benzyl­idene and hydrazinecarboxamide groups are oriented at a dihedral angle of 53.82 (6)° and an intra­molecular N—H⋯N hydrogen bond generates an *S*(5) ring motif. In the crystal, mol­ecules are linked by N—H⋯O hydrogen bonds into sheets propagating in (-201), which feature *R*_1_^2^(5), *R*_2_^2^(8) and *R*_2_^4^(14) loops.

## Related literature

For related structures, see: Fun *et al.* (2011 ▶); Liang *et al.* (2007 ▶); For graph-set notation, see: Bernstein *et al.* (1995 ▶).
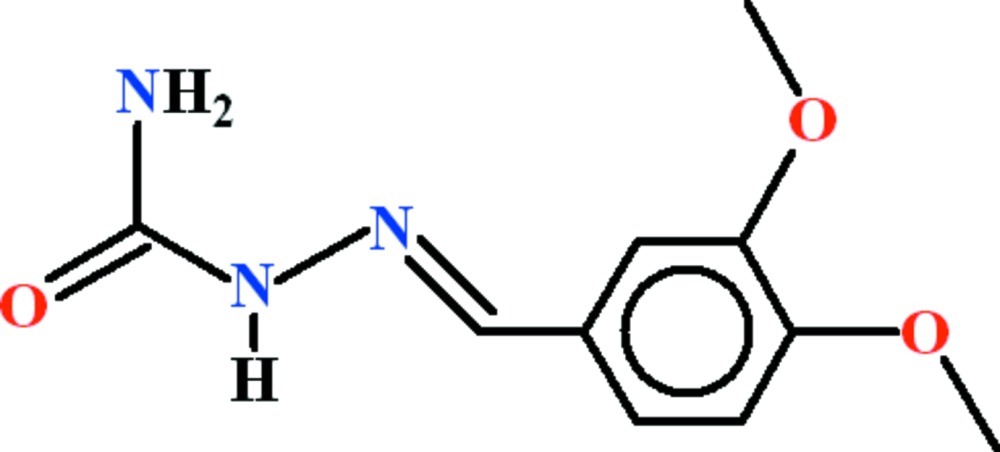


## Experimental

### 

#### Crystal data

C_10_H_13_N_3_O_3_
*M* *_r_* = 223.23
Monoclinic, 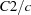

*a* = 22.2300 (7) Å
*b* = 7.6367 (3) Å
*c* = 15.6482 (6) Å
β = 126.234 (1)°
*V* = 2142.76 (14) Å^3^
*Z* = 8
Mo *K*α radiation
μ = 0.10 mm^−1^
*T* = 296 K
0.25 × 0.18 × 0.15 mm

#### Data collection

Bruker Kappa APEXII CCD diffractometer
Absorption correction: multi-scan (*SADABS*; Bruker, 2005 ▶) *T*_min_ = 0.975, *T*_max_ = 0.985
7933 measured reflections
2115 independent reflections
1389 reflections with *I* > 2σ(*I*)
*R*_int_ = 0.040

#### Refinement

*R*[*F* ^2^ > 2σ(*F* ^2^)] = 0.043
*wR*(*F* ^2^) = 0.121
*S* = 1.01
2115 reflections
147 parameters
H-atom parameters constrained
Δρ_max_ = 0.16 e Å^−3^
Δρ_min_ = −0.20 e Å^−3^

### 

Data collection: *APEX2* (Bruker, 2007 ▶); cell refinement: *SAINT* (Bruker, 2007 ▶); data reduction: *SAINT*; program(s) used to solve structure: *SHELXS97* (Sheldrick, 2008 ▶); program(s) used to refine structure: *SHELXL97* (Sheldrick, 2008 ▶); molecular graphics: *ORTEP-3 for Windows* (Farrugia, 1997 ▶) and *PLATON* (Spek, 2009 ▶); software used to prepare material for publication: *WinGX* (Farrugia, 1999 ▶) and *PLATON*.

## Supplementary Material

Crystal structure: contains datablock(s) global, I. DOI: 10.1107/S1600536812020739/hb6783sup1.cif

Structure factors: contains datablock(s) I. DOI: 10.1107/S1600536812020739/hb6783Isup2.hkl

Supplementary material file. DOI: 10.1107/S1600536812020739/hb6783Isup3.cml

Additional supplementary materials:  crystallographic information; 3D view; checkCIF report

## Figures and Tables

**Table 1:** Hydrogen-bond geometry (Å, °)

*D*—H⋯*A*	*D*—H	H⋯*A*	*D*⋯*A*	*D*—H⋯*A*
N2—H2*A*⋯O3^i^	0.86	2.15	2.970 (2)	159
N3—H3*A*⋯O3^ii^	0.86	2.19	3.044 (2)	170
N3—H3*B*⋯N1	0.86	2.30	2.657 (2)	105
N3—H3*B*⋯O1^iii^	0.86	2.59	3.019 (2)	112
N3—H3*B*⋯O2^iii^	0.86	2.30	3.119 (2)	160

Symmetry codes: (i) 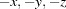; (ii) 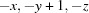; (iii) 
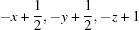
.

**Fig. 1.**
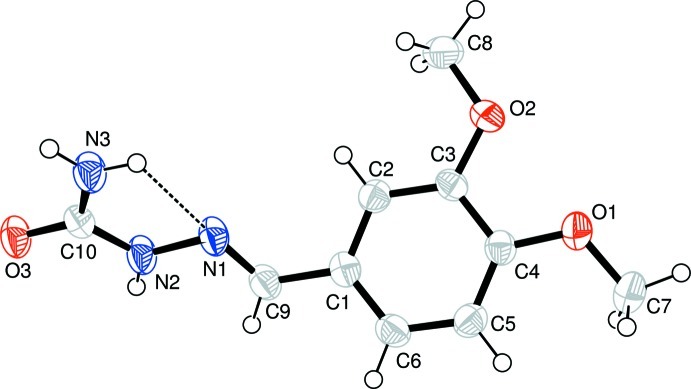
View of the title compound with displacement ellipsoids drawn at the 50% probability level. The dotted lines indicate the intra-molecular hydrogen bond.

**Fig. 2.**
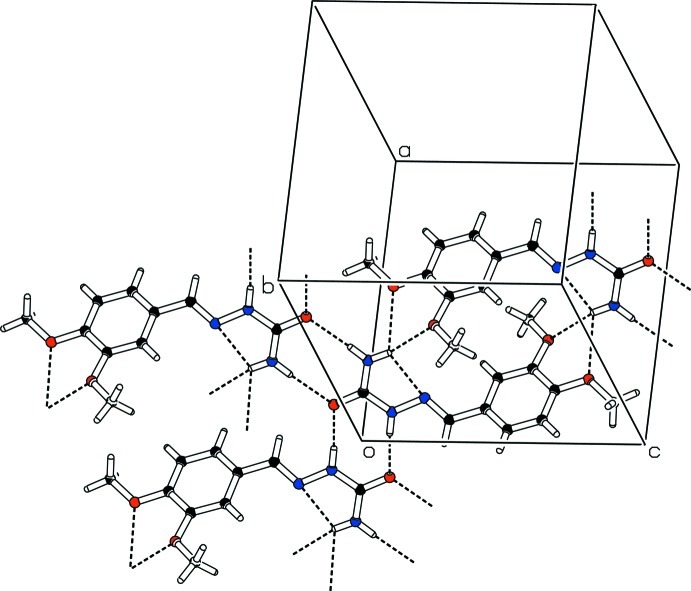
Partial packnig diagram showing molecules interlinked to form polymeric sheets with various ring motifs.

## supplementary crystallographic information

### Comment

The title compound (I), (Fig. 1) has been synthesized as a derivative.

The crystal structures of (*E*)-1-(4-methoxybenzylidene)semicarbazide (Liang *et al.*, 2007) and (*E*)-2-(4-hydroxy-3-methoxybenzylidene) hydrazinecarboxamide (Fun *et al.*, 2011) have been published which are related to the title compound (I).

In (I), the parts of 3,4-dimethoxybenzaldehyde and hydrazinecarboxamide A (C1—C9/O1/O2) and B (N1/N2/C10/N3/O3), are almost planar with r. m. s. deviation of 0.0770 and 0.0159 Å, respectively. The dihedral angle between A/B is 53.82 (6)°. There exist intramolecular H–bonding of N—H···N type (Table 1, Fig. 1) and form *S*(5) ring motif (Bernstein *et al.*, 1995). Each molecule is interlinked with three molecules due to H-bondings of N—H···O type. There exist *R*_1_^2^(5), *R*_2_^2^(8) and *R*_2_^4^(14) ring motifs (Table 1, Fig. 2). The molecules are interliked in the form of two-dimensional polymeric sheets in the plane (201) and with base vectors [100] and [102].

### Experimental

Equimolar quantities of 3,4-dimethoxybenzaldehyde and hydrazinecarboxamide were refluxed in methanol for 45 min resulting in yellow solution. The solution was kept at room temperature which affoarded yellow prisms after 48 h.

### Refinement

The H-atoms were positioned geometrically (C–H = 0.93–0.96 Å and N—H = 0.86 Å) and refined as riding with *U*_iso_(H) = x*U*_eq_(C. N), where x = 1.5 for methyl and x = 1.2 for all other H-atoms.

### Crystal data

**Table:**

C_10_H_13_N_3_O_3_	*F*(000) = 944
*M**_r_* = 223.23	*D*_x_ = 1.384 Mg m^−^^3^
Monoclinic, *C*2/*c*	Mo *K*α radiation, λ = 0.71073 Å
Hall symbol: -C 2yc	Cell parameters from 1389 reflections
*a* = 22.2300 (7) Å	θ = 2.3–26.0°
*b* = 7.6367 (3) Å	µ = 0.10 mm^−^^1^
*c* = 15.6482 (6) Å	*T* = 296 K
β = 126.234 (1)°	Prism, yellow
*V* = 2142.76 (14) Å^3^	0.25 × 0.18 × 0.15 mm
*Z* = 8	

### Data collection

**Table:**

Bruker Kappa APEXII CCD diffractometer	2115 independent reflections
Radiation source: fine-focus sealed tube	1389 reflections with *I* > 2σ(*I*)
Graphite monochromator	*R*_int_ = 0.040
Detector resolution: 8.00 pixels mm^-1^	θ_max_ = 26.0°, θ_min_ = 2.3°
ω scans	*h* = −26→27
Absorption correction: multi-scan (*SADABS*; Bruker, 2005)	*k* = −9→9
*T*_min_ = 0.975, *T*_max_ = 0.985	*l* = −19→19
7933 measured reflections	

### Refinement

**Table:**

Refinement on *F*^2^	Primary atom site location: structure-invariant direct methods
Least-squares matrix: full	Secondary atom site location: difference Fourier map
*R*[*F*^2^ > 2σ(*F*^2^)] = 0.043	Hydrogen site location: inferred from neighbouring sites
*wR*(*F*^2^) = 0.121	H-atom parameters constrained
*S* = 1.01	*w* = 1/[σ^2^(*F*_o_^2^) + (0.0592*P*)^2^ + 0.0283*P*] where *P* = (*F*_o_^2^ + 2*F*_c_^2^)/3
2115 reflections	(Δ/σ)_max_ < 0.001
147 parameters	Δρ_max_ = 0.16 e Å^−^^3^
0 restraints	Δρ_min_ = −0.20 e Å^−^^3^

### Special details

**Table:**

Geometry. Bond distances, angles *etc*. have been calculated using the rounded fractional coordinates. All su's are estimated from the variances of the (full) variance-covariance matrix. The cell e.s.d.'s are taken into account in the estimation of distances, angles and torsion angles
Refinement. Refinement of *F*^2^ against ALL reflections. The weighted *R*-factor *wR* and goodness of fit *S* are based on *F*^2^, conventional *R*-factors *R* are based on *F*, with *F* set to zero for negative *F*^2^. The threshold expression of *F*^2^ > σ(*F*^2^) is used only for calculating *R*-factors(gt) *etc*. and is not relevant to the choice of reflections for refinement. *R*-factors based on *F*^2^ are statistically about twice as large as those based on *F*, and *R*- factors based on ALL data will be even larger.

### Fractional atomic coordinates and isotropic or equivalent isotropic displacement parameters (Å^2^)

**Table:**

	*x*	*y*	*z*	*U*_iso_*/*U*_eq_	
O1	0.30582 (7)	−0.13305 (19)	0.72476 (10)	0.0464 (5)	
O2	0.34785 (7)	0.03543 (19)	0.62646 (10)	0.0454 (5)	
O3	−0.01303 (7)	0.24827 (17)	−0.02345 (10)	0.0447 (5)	
N1	0.10093 (8)	0.0967 (2)	0.23838 (12)	0.0404 (5)	
N2	0.04892 (8)	0.0982 (2)	0.12929 (12)	0.0421 (5)	
N3	0.06302 (9)	0.3952 (2)	0.13090 (13)	0.0445 (6)	
C1	0.15297 (10)	−0.0591 (2)	0.40077 (15)	0.0347 (6)	
C2	0.22548 (10)	0.0085 (2)	0.45703 (15)	0.0356 (6)	
C3	0.27503 (10)	−0.0179 (2)	0.56479 (14)	0.0331 (6)	
C4	0.25221 (10)	−0.1097 (2)	0.61917 (15)	0.0345 (6)	
C5	0.18023 (10)	−0.1725 (3)	0.56366 (15)	0.0394 (7)	
C6	0.13153 (10)	−0.1495 (3)	0.45538 (15)	0.0397 (7)	
C7	0.28984 (13)	−0.2490 (3)	0.78007 (16)	0.0527 (8)	
C8	0.37782 (11)	0.1099 (3)	0.57544 (17)	0.0490 (8)	
C9	0.10014 (10)	−0.0369 (3)	0.28603 (15)	0.0400 (7)	
C10	0.03155 (9)	0.2502 (3)	0.07501 (15)	0.0347 (6)	
H2	0.24028	0.07157	0.42152	0.0427*	
H2A	0.02764	0.00235	0.09601	0.0505*	
H3A	0.05296	0.49443	0.09922	0.0534*	
H3B	0.09351	0.38993	0.19885	0.0534*	
H5	0.16442	−0.23070	0.59936	0.0473*	
H6	0.08359	−0.19543	0.41857	0.0476*	
H7A	0.24962	−0.20238	0.77979	0.0791*	
H7B	0.33332	−0.26156	0.85185	0.0791*	
H7C	0.27580	−0.36131	0.74591	0.0791*	
H8A	0.35195	0.21675	0.54077	0.0735*	
H8B	0.37181	0.02896	0.52397	0.0735*	
H8C	0.42987	0.13423	0.62723	0.0735*	
H9	0.06472	−0.12360	0.24658	0.0479*	

### Atomic displacement parameters (Å^2^)

**Table:**

	*U*^11^	*U*^22^	*U*^33^	*U*^12^	*U*^13^	*U*^23^
O1	0.0482 (8)	0.0532 (9)	0.0289 (8)	−0.0080 (7)	0.0179 (7)	0.0017 (7)
O2	0.0369 (8)	0.0576 (9)	0.0338 (8)	−0.0152 (7)	0.0165 (7)	−0.0050 (7)
O3	0.0471 (8)	0.0416 (9)	0.0264 (7)	0.0006 (6)	0.0113 (7)	−0.0004 (6)
N1	0.0408 (9)	0.0384 (10)	0.0272 (9)	0.0008 (7)	0.0120 (8)	−0.0009 (7)
N2	0.0448 (10)	0.0355 (9)	0.0263 (9)	−0.0042 (8)	0.0102 (8)	−0.0018 (7)
N3	0.0476 (10)	0.0351 (10)	0.0320 (10)	−0.0030 (8)	0.0132 (8)	−0.0010 (7)
C1	0.0362 (10)	0.0307 (10)	0.0330 (11)	0.0031 (8)	0.0181 (9)	−0.0009 (8)
C2	0.0401 (11)	0.0327 (11)	0.0345 (11)	−0.0023 (8)	0.0224 (9)	−0.0013 (8)
C3	0.0321 (10)	0.0330 (10)	0.0303 (11)	−0.0042 (8)	0.0163 (9)	−0.0062 (8)
C4	0.0374 (10)	0.0335 (10)	0.0304 (10)	0.0001 (8)	0.0188 (9)	−0.0022 (8)
C5	0.0417 (11)	0.0418 (12)	0.0394 (12)	−0.0001 (9)	0.0266 (10)	0.0031 (9)
C6	0.0307 (10)	0.0406 (12)	0.0421 (12)	0.0005 (9)	0.0184 (9)	0.0015 (9)
C7	0.0604 (14)	0.0619 (16)	0.0364 (12)	−0.0022 (11)	0.0289 (11)	0.0071 (11)
C8	0.0462 (12)	0.0524 (14)	0.0524 (14)	−0.0126 (10)	0.0313 (12)	−0.0047 (11)
C9	0.0350 (11)	0.0397 (12)	0.0337 (11)	−0.0015 (9)	0.0140 (10)	−0.0014 (9)
C10	0.0289 (10)	0.0391 (11)	0.0294 (10)	−0.0001 (8)	0.0136 (9)	−0.0010 (9)

### Geometric parameters (Å, º)

**Table:**

O1—C4	1.362 (2)	C2—C3	1.379 (3)
O1—C7	1.422 (3)	C3—C4	1.408 (3)
O2—C3	1.368 (3)	C4—C5	1.379 (3)
O2—C8	1.426 (3)	C5—C6	1.380 (3)
O3—C10	1.245 (2)	C2—H2	0.9300
N1—N2	1.385 (2)	C5—H5	0.9300
N1—C9	1.270 (3)	C6—H6	0.9300
N2—C10	1.353 (3)	C7—H7A	0.9600
N3—C10	1.326 (3)	C7—H7B	0.9600
N2—H2A	0.8600	C7—H7C	0.9600
N3—H3A	0.8600	C8—H8A	0.9600
N3—H3B	0.8600	C8—H8B	0.9600
C1—C9	1.463 (3)	C8—H8C	0.9600
C1—C2	1.401 (3)	C9—H9	0.9300
C1—C6	1.384 (3)		
			
C4—O1—C7	117.75 (18)	N2—C10—N3	117.33 (17)
C3—O2—C8	118.26 (15)	O3—C10—N2	119.32 (19)
N2—N1—C9	116.05 (17)	C1—C2—H2	120.00
N1—N2—C10	120.10 (15)	C3—C2—H2	120.00
N1—N2—H2A	120.00	C4—C5—H5	120.00
C10—N2—H2A	120.00	C6—C5—H5	120.00
C10—N3—H3A	120.00	C1—C6—H6	120.00
C10—N3—H3B	120.00	C5—C6—H6	120.00
H3A—N3—H3B	120.00	O1—C7—H7A	109.00
C2—C1—C6	118.95 (18)	O1—C7—H7B	109.00
C2—C1—C9	121.3 (2)	O1—C7—H7C	109.00
C6—C1—C9	119.7 (2)	H7A—C7—H7B	109.00
C1—C2—C3	120.4 (2)	H7A—C7—H7C	109.00
C2—C3—C4	119.9 (2)	H7B—C7—H7C	109.00
O2—C3—C4	114.88 (16)	O2—C8—H8A	109.00
O2—C3—C2	125.2 (2)	O2—C8—H8B	109.00
O1—C4—C5	125.2 (2)	O2—C8—H8C	109.00
O1—C4—C3	115.4 (2)	H8A—C8—H8B	109.00
C3—C4—C5	119.39 (18)	H8A—C8—H8C	109.00
C4—C5—C6	120.3 (2)	H8B—C8—H8C	109.00
C1—C6—C5	121.0 (2)	N1—C9—H9	119.00
N1—C9—C1	122.29 (19)	C1—C9—H9	119.00
O3—C10—N3	123.4 (2)		
			
C7—O1—C4—C3	−169.75 (18)	C2—C1—C9—N1	−31.7 (3)
C7—O1—C4—C5	8.3 (3)	C6—C1—C9—N1	148.8 (2)
C8—O2—C3—C2	−6.2 (3)	C1—C2—C3—O2	176.97 (18)
C8—O2—C3—C4	172.38 (17)	C1—C2—C3—C4	−1.5 (3)
C9—N1—N2—C10	162.2 (2)	O2—C3—C4—O1	−0.4 (2)
N2—N1—C9—C1	178.4 (2)	O2—C3—C4—C5	−178.58 (18)
N1—N2—C10—O3	177.1 (2)	C2—C3—C4—O1	178.23 (17)
N1—N2—C10—N3	−3.6 (3)	C2—C3—C4—C5	0.1 (3)
C6—C1—C2—C3	1.3 (3)	O1—C4—C5—C6	−176.4 (2)
C9—C1—C2—C3	−178.29 (19)	C3—C4—C5—C6	1.6 (3)
C2—C1—C6—C5	0.4 (3)	C4—C5—C6—C1	−1.9 (3)
C9—C1—C6—C5	180.0 (2)		

### Hydrogen-bond geometry (Å, º)

**Table:**

*D*—H···*A*	*D*—H	H···*A*	*D*···*A*	*D*—H···*A*
N2—H2*A*···O3^i^	0.86	2.15	2.970 (2)	159
N3—H3*A*···O3^ii^	0.86	2.19	3.044 (2)	170
N3—H3*B*···N1	0.86	2.30	2.657 (2)	105
N3—H3*B*···O1^iii^	0.86	2.59	3.019 (2)	112
N3—H3*B*···O2^iii^	0.86	2.30	3.119 (2)	160

Symmetry codes: (i) −*x*, −*y*, −*z*; (ii) −*x*, −*y*+1, −*z*; (iii) −*x*+1/2, −*y*+1/2, −*z*+1.
